# Supplementary material for: Acetylated α-Tubulin and α-Synuclein: Physiological Interplay and Contribution to α-Synuclein Oligomerization
Source: Int J Mol Sci. 2023 Jul 31;24(15):12287. doi: 10.3390/ijms241512287 (PMC10418364; doi:10.3390/ijms241512287)
Supplement: Supplementary file 1 [file ijms-24-12287-s001.zip › ijms-2480997-supplementary.pdf]

Supplementary Materials: The following supporting information can be downloaded at: www.mdpi.com/xxx/s1, Supplementary file.

Acetylated α-tubulin and α-synuclein: physiological interplay and contribution to α-synuclein oligomerization.

Alessandra Maria Calogero[1,2,*], Milo Jarno Basellini[1], Huseyin Berkcan Isilgan[1], Francesca Longhena[3], Arianna Bellucci[3], Samanta Mazzetti[1,2], Chiara Rolando[1], Gianni Pezzoli[2,4] and Graziella Cappelletti[1,5,*]

1 Department of Biosciences, Università degli Studi di Milano, Milan, Italy; alessandra.calogero@unimi.it (A.M.C.); milo.basellini@unimi.it (M.J.B.); huseyin.isilgan@unimi.it (H.B.I.); samanta.mazzetti@gmail.com (S.M.); chiara.rolando@unimi.it (C.R.); graziella.cappelletti@unimi.it (G.C.)

2 Fondazione Grigioni per il Morbo di Parkinson, Milan, Italy; alessandra.calogero@unimi.it (A.M.C.); samanta.mazzetti@gmail.com (S.M.); pezzoli@parkinson.it (G.P.)

3 Department of Molecular and Translational Medicine, University of Brescia, Brescia, Italy; f.longhena@unibs.it (F.L.); arianna.bellucci@unibs.it (A.B.)

4 Parkinson Institute, ASST-Pini-CTO, Milan, Italy; pezzoli@parkinson.it (G.P.)

5 Center of Excellence on Neurodegenerative Diseases, Università degli Studi di Milano, Milan, Italy; graziella.cappelletti@unimi.it (G.C.)

* Correspondence: graziella.cappellletti@unimi.it (G.C.); +39 02503 14752; alessandra.calogero@unimi.it (A.M.C.); +39 02503 14752

Supplementary Figures:

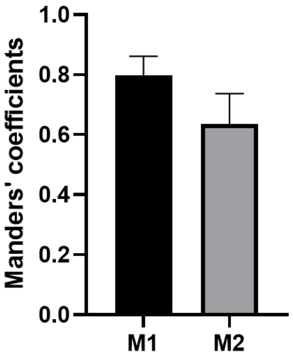

Figure S1. Manders' coefficients of α-synuclein and acetylated α-tubulin.

Graph represents the Manders' coefficient values expressing the colocalization of α-synuclein and acetylated α-tubulin in primary midbrain neurons. Manders' coefficient M1 (0.798 ± 0.063) represents the portion of α-synuclein overlapping to acetylated α-tubulin. Manders' coefficient M2 (0.636 ± 0.101) represents the portion of acetylated α-tubulin overlapping with α-synuclein. N=4 biological replicates, 26 cells. Values are expressed as mean ± standard error of the mean.

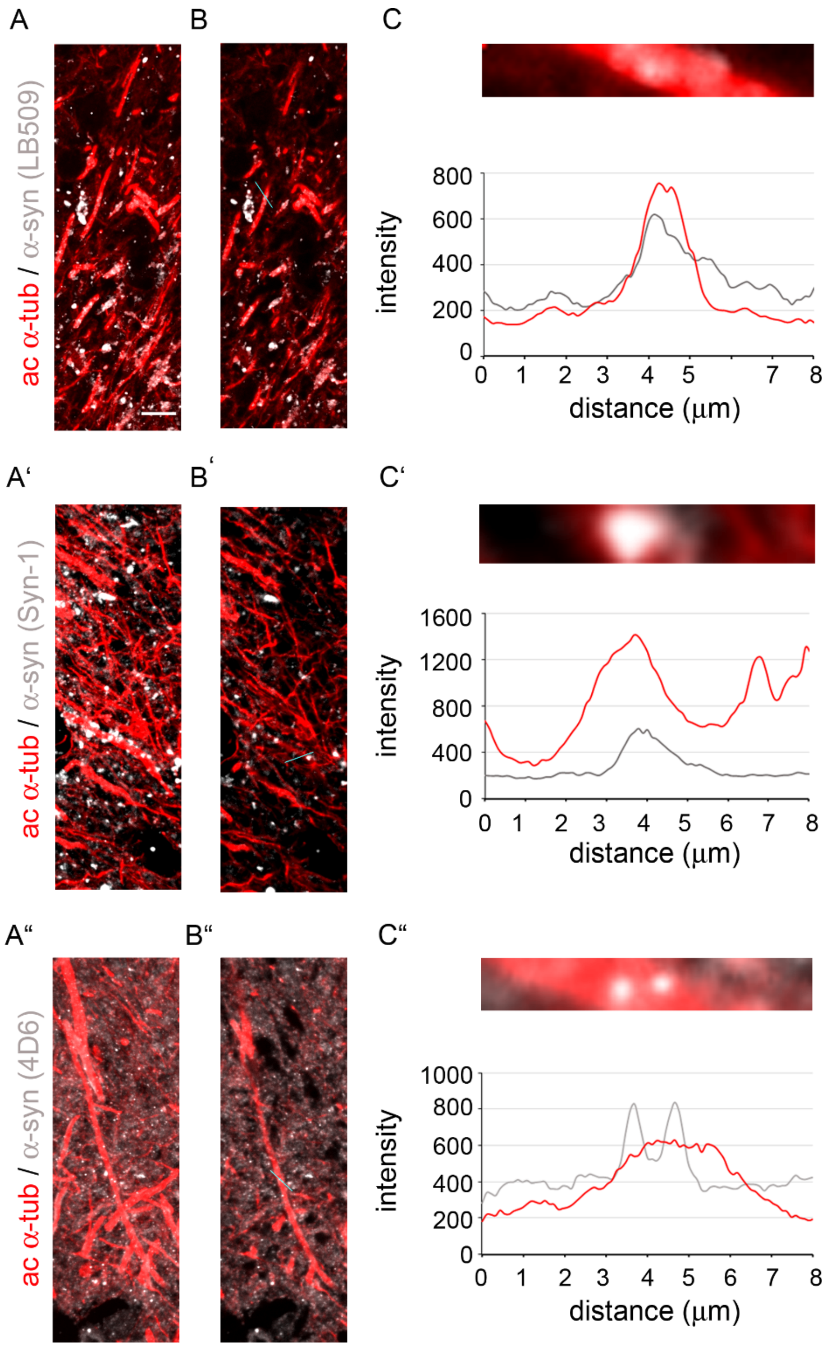

Figure S2. α-Synuclein localization on acetylated microtubules using different anti α-synuclein antibodies.

Confocal microscopy images of *substantia nigra* showing the distribution of acetylated α-tubulin (ac α-tub, in red) and α-synuclein using three different antibodies against α-synuclein (white): anti α-synuclein LB509 (A-C), anti α-synuclein Syn-1 (A'-C') anti α-synuclein 4D6 (A"-C"). (A, A', A") Maximum projection. Scale bar: 10 µm. (B, B', B") Single optical sections, depth: 0.3 µm. (C, C', C") Detail of a portion of acetylated microtubules in which α-synuclein is present (cyan lines in B, B', B") and relative intensity profile of the two signals in the single optical section showed in (B, B', B").

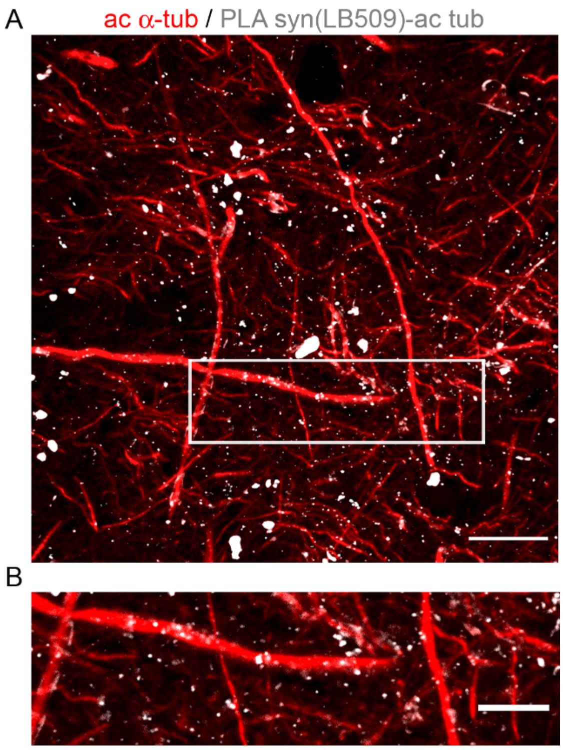

Figure S3. Validation of α-synuclein close proximity with acetylated α-tubulin.

(A) Representative confocal image of *substantia nigra* of *post-mortem* human brain showing the presence of the *in situ* Proximity Ligation Assay (PLA) signal obtained using the anti α-synuclein antibody LB509 and the anti acetylated α-tubulin D20G3. The presence of PLA staining with this couple of antibodies confirms the PLA data obtained with the anti α-synuclein antibody S3062 and the anti acetylated α-tubulin 6-11 B-1 (shown Figure 3). Scale bar: 20 µm. (B) Zoomed detail of the inset in A. Scale bar: 10 µm.

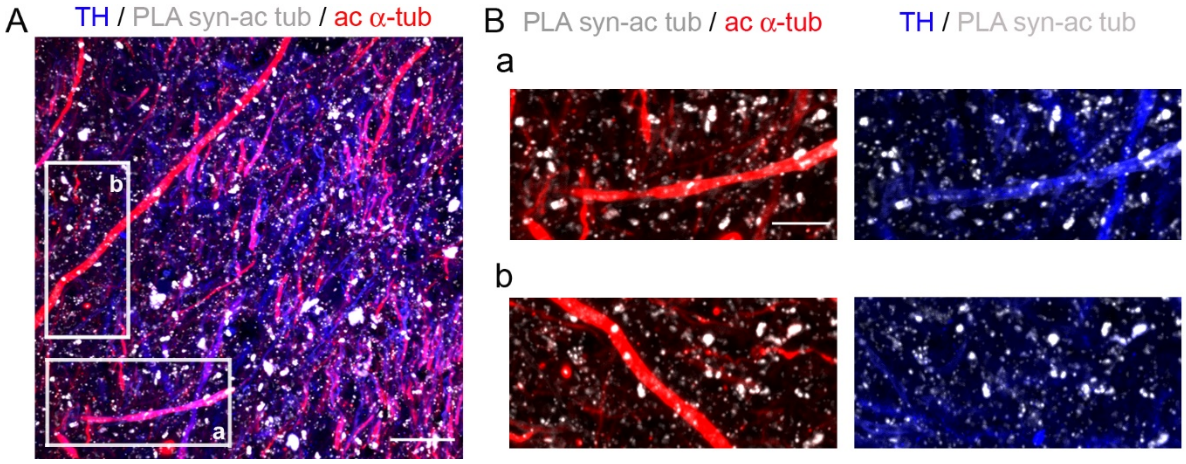

Figure S4. Analysis of α-Synuclein and acetylated α-tubulin closed proximity in dopaminergic and not dopaminergic neurons.

(A) Representative confocal image of *substantia nigra* of *post-mortem* human brain showing the presence of the *in situ* Proximity Ligation Assay (PLA) signal between α-synuclein and acetylated α-tubulin (PLA syn-ac tub, white), and the immunofluorescence staining of acetylated α-tubulin (ac α-tub, red) and tyrosine hydroxylase (TH, blue). Scale bar: 20 µm. (B) Zoomed details of the insets in A. The PLA staining is clearly detectable along acetylated microtubules (red) in both TH-positive neurons (a) and in TH-negative neurons (b). Scale bar: 10 µm.

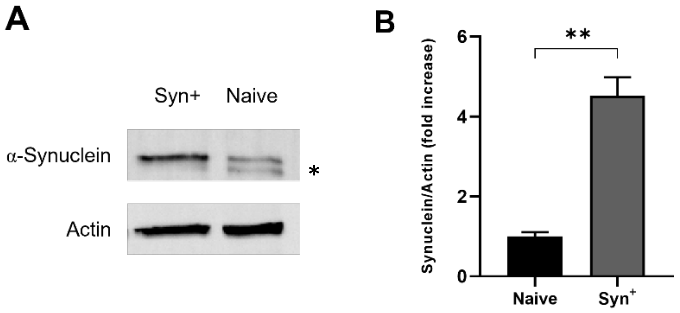

Figure S5. α-Synuclein overexpression in SK-N-SH Syn$^+$.

(A) Representative western blotting showing the expression of α-synuclein and actin in SK-N-SH Naïve (Naïve) and α-synuclein stable transfected SK-N-SH (Syn$^+$) cells. (B) Quantification of α-synuclein levels (relative abundance) expressed as fold change relative to naive cells. N=3 biological replicates, ** $p < 0.005$ according to Student's t-test.

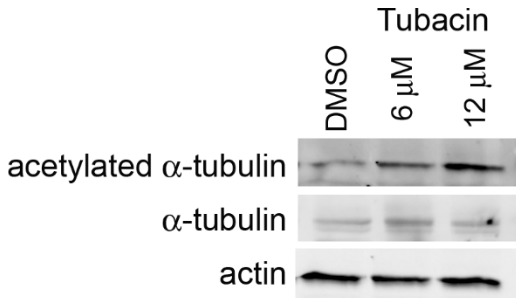

Figure S6. Tubacin treatment induces an increase in acetylated α-tubulin in SK-N-SH Syn$^+$.

Western blotting of SK-N-SH Syn$^+$ cells treated with DMSO (vehicle) or tubacin (6 µM and 12 µM) showing the increase in acetylated α-tubulin caused by Tubacin treatment.

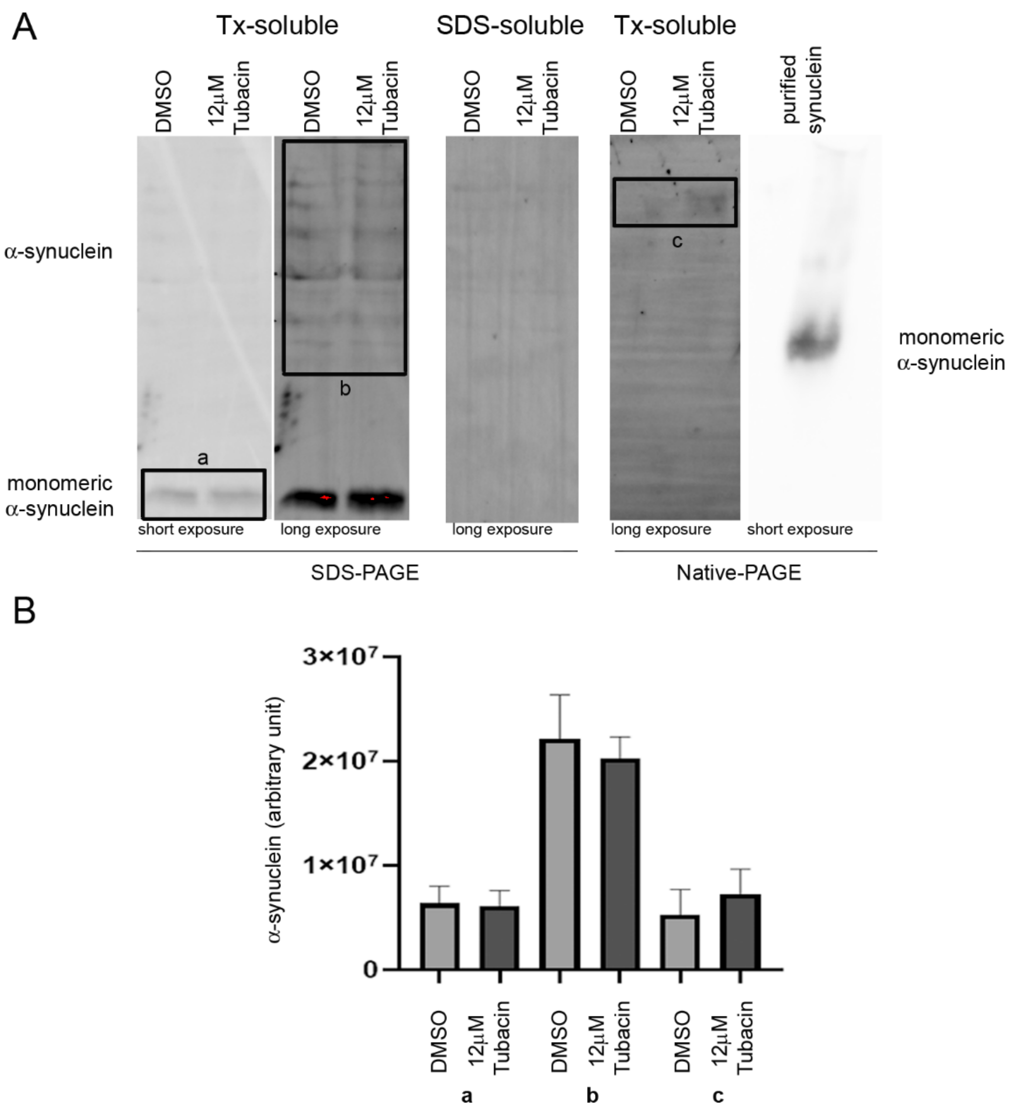

Figure S7. Biochemical fractionation of SK-N-SH Syn$^+$.

(A) Representative images of Western blotting representing Tx-soluble and SDS-soluble fractions of SK-N-SH Syn$^+$ following treatment with 12 µM Tubacin or vehicle (DMSO). Equal amounts of extracts were loaded in SDS-PAGE or Native-PAGE to evaluate the presence of different species of α-synuclein. Images obtained following short and long exposures are showed. Monomeric α-synuclein (a) is visible following short exposure, whereas long exposures are required to highlight bands that could correspond to multimers of α-synuclein (b) in the Tx-soluble fraction loaded in SDS-PAGE. Red pixels in the long exposure panel indicate saturated signals. In the SDS-soluble fractions no staining is detectable at long exposure time, indicating the absence of big aggregates of α-synuclein, as expected. In the Native-PAGE, faint signals can be detectable in the upper part of the gel and could indicate the presence of oligomeric species of α-synuclein (c) since the signal of the monomeric α-synuclein is detectable in the lower part of this gel. 0.5 µg of monomeric purified human α-synuclein was loaded as migration control. (B) Quantification of the levels of monomeric α-

synuclein (a) and high molecular weight species of α-synuclein (b) in Tx-soluble fractions loaded on SDS-PAGE and the levels of α-synuclein in the upper part of Native-PAGE (c) are shown in the graph. N=3 biological replicates.

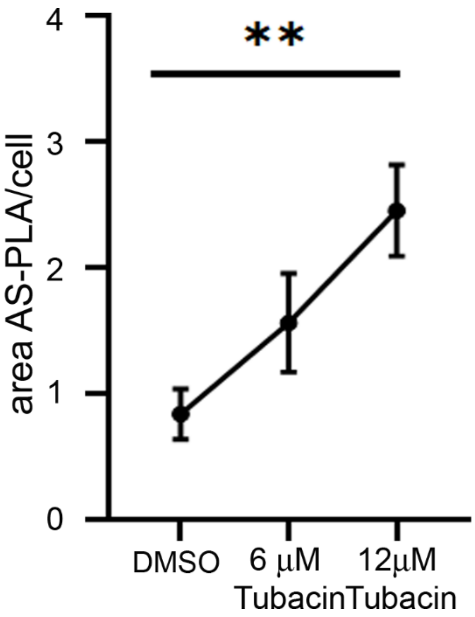

Figure S8. Tubacin treatment induces an increase in oligomeric α-synuclein in SK-N-SH Syn⁺.

Graph represents the increase of oligomeric α-synuclein expressed as the area of AS-PLA per cell in SK-N-SH Syn^+ cells treated with DMSO (vehicle) or tubacin (6 μM and 12 μM). N=4 biological replicates, **p=0.0046 according to One way ANOVA, Friedman test.

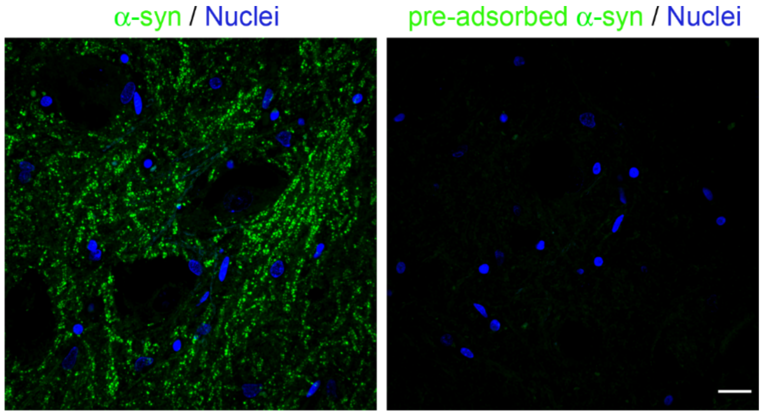

Figure S9. Pre-adsorption of anti α-synuclein S3062 antibody.

Sections of *substantia nigra* incubated with anti α-synuclein antibody (S3062) alone or pre-adsorbed with human α-synuclein. The specific staining for α-synuclein (green) is undetectable in sections incubated with pre-adsorbed antibody. Nuclei are counterstained with Hoechst. Scale bar: 20 μm.

Supplementary Table:

Table S1. List of primary and secondary antibodies.

Primary antibody					
**Name**	**Dilution**	**Application**	**host**	**Cat ID**	**brand**
anti α-Synuclein	1:500<br>1:2000<br>1:1000	IF cells<br>IF human brain<br>PLA human brain	Rabbit	S3062	Sigma-Aldrich
anti α-Synuclein (clone LB 509)	1:500<br>1:250	IF human brain<br>PLA human brain	Mouse	AB27766	Abcam
anti α-Synuclein (clone 4D6)	1:1000	IF human brain	Mouse	SIG-39720	Covance
anti α-Synuclein (Syn-1)	1:50	IF human brain	Mouse	610787	BD Biosciences
anti α-Synuclein conjugated to MINUS or PLUS probes	1:100	AS-PLA	Rabbit	S3062	Sigma-Aldrich
anti acetylated α-tubulin (clone 6-11 B-1)	1:300<br>1:150	IF<br>PLA human brain	Mouse	T7451	Sigma-Aldrich
anti acetylated α-tubulin (clone D20G3)	1:200<br>1:100	IF human brain<br>PLA human brain	Rabbit	#5335	Cell Signalling Technology
Tyrosine hydroxylase	1:200	IF human brain	Goat	PA5-18372	Invitrogen
**Secondary antibody**					
**Name**	**Dilution**		**host**	**Cat ID**	**Brand**
anti rabbit IgG (H+L) Alexa Fluor™ 488	1:1000<br>1:200	IF cells<br>IF human brain	Donkey	A21206	Invitrogen
anti mouse Alexa IgG (H+L) Alexa Fluor™ 568	1:1000<br>1:200	IF cells<br>IF human brain	Donkey	A10037	Invitrogen
anti mouse IgG (H+L) Alexa Fluor™ 488	1:1000	IF cells	Donkey	A21202	Invitrogen
anti rabbit IgG (H+L) Alexa Fluor™ Plus 647	1:1000	IF cells	Donkey	A32795	Invitrogen
anti mouse IgG (H+L) Alexa Fluor™ Plus 647	1:200	IF human brain	Donkey	A32787	Invitrogen
anti goat Brilliant Violet 421	1:300	IF human brain	Donkey	705-675-147	Jackson ImmunoResearch Europe LTD

IF: immunofluorescence assay; PLA: Proximity Ligation Assay; AS-PLA: alpha Synuclein Proximity Ligation Assay

Supplementary Materials and Methods:

Pre-adsorption of anti α-synuclein S3062 antibody

The specificity of the anti α-synuclein antibody made in rabbit (S3062) used in this work was verified by pre-adsorption with the recombinant human α-synuclein protein. The protein was purified according to [71] and as previously reported [9, 72]. Briefly, pET-21d(+) plasmid coding for human α-synuclein was expressed in BL21DE3 *E.coli*. Once the bacterial cultures reached the midlog phase, 0.4mM IPTG was added. After 2 hours at 37°C in agitation, cells were pelleted, washed in PBS, resuspended in HEPES buffer (20 mM HEPES, 100 mM KCl, pH 7.2) and finally heated at 90°C for 10 minutes. Aggregated proteins were removed by centrifugation (40 minutes at 40,000xg at 4 °C). Supernatant containing α-synuclein was loaded in a Q-Sepharose Hi-Trap column (Amersham Biosciences) equilibrated with Solution A (50 mM Tris pH 7.4) to remove contaminating nucleic acids and proteins by ion exchange chromatography. Purified α-synuclein was eluted applying an increasing gradient of Solution B (50 mM Tris pH 7.4, 1M KCl). Eluted fraction containing α-synuclein were pooled, chromatographed, and desalted on a PD Midi Trap G25 (GE Healthcare Bio-Sciences AB) in milliQ water to be lyophilized and stored at -80°C until use. For pre-adsorption assay, lyophilized α-synuclein was resuspended in PBS. A solution containing anti α-synuclein antibody or both anti α-synuclein antibody and purified α-synuclein protein (1:5) in 1% BSA in PBS were incubated in agitation ON at RT. The day after, the two mixtures were used to perform immunofluorescence assay on *post-mortem* human brain samples (Figure S9) as previously described.

Western blotting

Total cellular extracts were obtained using Lysis buffer SB1X (2% SDS, 10% glycerol, 5% β-mercaptoethanol, 0.001% bromophenol blue, and 62.5 mmol/L Tris, pH 6.8) in the presence of protease (M250, AMRESCO) and phosphatase inhibitors (Phosphatase Inhibitor Cocktail Set V, 524629, Millipore). Briefly, cells were washed twice with PBS and scraped into SB1X. Protein concentration was measured with Micro BCA™ Protein Assay Kit (Thermo Scientific) and equal amounts of each sample were separated by SDS-PAGE. After electrophoresis, proteins were blotted onto PVDF membrane (Immobilon-F transfer membrane, Millipore), for 1.5 hours at 60V, 4°C. Membranes were blocked with 5% BSA, 0.05% Tween in Tris-buffered saline (TBS) for 1 hour at RT and then incubated ON at 4°C with the following primary antibodies: anti α-synuclein rabbit IgG antibody (1:1000; S3062, Sigma), anti acetylated α-tubulin mouse IgG antibody (1:2000; clone 6-11B-1, Sigma) anti α-tubulin antibody (1:2000; T6074, Sigma), anti actin rabbit IgG antibody (1:2000; A2066, Sigma). The incubation with secondary antibodies was performed for 1 hour at RT,

in the dark, using the following Alexa-conjugated antibodies: Alexa Fluor 568 donkey anti mouse (1:4000; Invitrogen, A10037); Alexa Fluor 488 donkey anti rabbit (1:4000; Invitrogen, A21206); Alexa Fluor Plus 488 goat anti mouse (1:5000; A32723; Invitrogen), diluted in in BSA 1% in TBS + Tween 0.1%. Acquisition and quantification were performed by Chemidoc and Image Lab software (Bio-Rad).

Biochemical fractionation

To evaluate the presence of oligomeric forms of α-synuclein with a biochemical approach, SK-N-SH Syn^+ cells treated with DMSO or Tubacin were analysed according to the biochemical fractionation protocol reported in [73]. Briefly, cells were lysed in STET buffer (150 mM NaCl, 50 mM Tris pH 7.6, 1% Triton X-100, 2 mM EDTA) supplemented with protease and phosphatase inhibitors, incubated for 30 minutes at 4°C and centrifugated at 13000*xg* for 30 minutes at 4°C. The supernatants, corresponding to the Tx-soluble fractions, were collected and the protein concentrations measured. The remaining pellets (SDS-soluble fractions) were washed twice with ice-cold PBS, resuspended in 2% SDS buffer (150 mM NaCl, 50 mM Tris pH 7.6, 2% SDS, 2 mM EDTA, supplemented with protease and phosphatase inhibitors), incubated for 15 minutes at 25°C and finally centrifugated. After addition of proper samples buffers for SDS-PAGE, the Tx-soluble fraction was incubated at 95°C for 5 minutes, while the SDS-soluble fraction at 42°C for 20 minutes. For Native-PAGE, the Tx-soluble fraction was resuspended in 4x Native sample buffer (250 mM Tris pH 6.8, 40% Glycerol; 0.04 bromophenol blue). The same amount of protein lysates was loaded on SDS-PAGE or Native-PAGE, and electrophoresis and blotting performed as previously reported. Blotted membranes were treated with 4% PFA in PBS for 30 minutes at RT in agitation, according to [74] and after two washes, blocked with 5% BSA, 0.05% Tween in TBS and incubated ON at 4°C with the anti α-synuclein rabbit IgG antibody as previously indicated.

References

71. Martinez, J.; Moeller, I.; Erdjument-Bromage, H.; Tempst, P.; Lauring, B. Parkinson's Disease-Associated α-Synuclein Is a Calmodulin Substrate. Journal of Biological Chemistry 2003, 278, 17379–17387, doi:10.1074/jbc.M209020200.
72. Baden, P.; Perez, M.J.; Raji, H.; Bertoli, F.; Kalb, S.; Illescas, M.; Spanos, F.; Giuliano, C.; Calogero, A.M.; Oldrati, M.; et al. Glucocerebrosidase Is Imported into Mitochondria and Preserves Complex I Integrity and Energy Metabolism. *Nat Commun* **2023**, *14*, 1930, doi:10.1038/s41467-023-37454-4.
73. Pantazopoulou, M.; Brembati, V.; Kanellidi, A.; Bousset, L.; Melki, R.; Stefanis, L. Distinct Alpha-Synuclein Species Induced by Seeding Are Selectively Cleared by the Lysosome or

the Proteasome in Neuronally Differentiated SH-SY5Y Cells. J Neurochem 2021, 156, 880–896, doi:10.1111/jnc.15174.
74. Sasaki, A.; Arawaka, S.; Sato, H.; Kato, T. Sensitive Western Blotting for Detection of Endogenous Ser129-Phosphorylated α-Synuclein in Intracellular and Extracellular Spaces. *Sci Rep* 2015, *5*, 14211, doi:10.1038/srep14211.
